# Protein Expressions and Their Immunogenicity from *Riemerella anatipestifer* Cultured in Iron Restriction Medium

**DOI:** 10.1371/journal.pone.0065901

**Published:** 2013-06-03

**Authors:** Yifei Yang, Changqin Gu, Yonghong Liao, Qingping Luo, Xueying Hu, Wanpo Zhang, Huabin Shao, Guofu Cheng

**Affiliations:** 1 College of Veterinary Medicine, Huazhong Agricultural University, Wuhan, China; 2 Institute of Animal Science, Academy of Agricultural Science of Hubei Province, Wuhan, China; 3 College of Veterinary Medicine, China Agricultural University, Beijing, China; Universidad Nacional de La Plata., Argentina

## Abstract

*Riemerella anatipestifer* was cultured in both iron restriction media and normal media. Two-dimensional gel electrophoresis identified 23 proteins that significantly increased in the iron restriction media. Of them 12 proteins were analyzed with mass spectrography. Nine of 12 proteins belong to 6 different protein families: fibronectin type iii domain protein, secreted subtilase family protein, phosphoglycerate kinase, translation elongation factor, leucine-rich repeat-containing protein, and Galactose-binding domain-like protein. Other 3 proteins were novel with unknown function. Two novel proteins (Riean_1750 and Riean_1752) were expressed in prokaryotic expression systems. The specificities of these 2 novel proteins to *R. anatipestifer* were confirmed by western-blotting analysis. The ducks immunized with either protein had low mortality challenged by *R. anatipestifer*, 33.3% and 16.7%, respectively. The ducks developed 100% immunity when immunized with combined Riean_1750 and Riean_1752 proteins. The data suggested 2 novel proteins play important roles in the bacterial survival in the iron restricted environment. They could be used as subunit vaccines of *R. anatipestifer*.

## Introduction

*Riemerella anatipestifer* (R. *anatipestifer*) is a Gram-negative, non-motile, non-spore-forming, rod-shaped bacterium, belongs to the Flavobacteriaceae family in rRNA superfamily V [1]. *R. anatipestifer* causes a contagious septicemia [2], which is characterized by fibrinous pericarditis, perihepatitis, and airsacculitis. Riemerellosis is a widely distributed disease that primarily affects young ducks and turkeys, but it has also been reported in other waterfowls, chickens, and pheasants [3]. Riemerellosis can cause significant economic losses in duck farmers [2], [4], especially in China and Southeastern Asia [5]–[7].

Iron is one of essential nutrients for the survival of both bacteria and hosts [8], [9]. To combat between hosts and pathogens for iron acquisition, the hosts have developed mechanisms to prevent bacterial growth by withhold iron from pathogens, while bacteria have the capacity to adapt to iron restricted environment by expression a large numbers of genes that up-regulate the uptake of iron from the hosts[10], [11]. In some Gram-positive, siderophore is one of these gene products produced in the environment of iron deficiency and forms the strongest soluble ferric-siderophore complexes that can be taken up by active transport mechanisms [10]. Because of this property, siderophores have attracted high attentions in the prevention and treatments of bacterial infectious diseases [10], [12].

The gene expressions of *R. anatipestifer* at the condition of iron deficiency are not completely clear, although the complete genome of *R. anatipestifer* has been sequenced and its immunoproteomics were reported [13]–[15]. To better understand its survival mechanisms in the environment of iron depletion, *R. anatipestifer* was cultured in either iron restricted or normal medium. The secreted proteins were compared by two-dimensional polyacrylamide gel electrophoresis (2-D PAGE). The proteins expressed at significantly higher levels in the iron restricted medium were identified for mass spectrometry (MS) analysis. The genes of these selected proteins were cloned and expressed in a prokaryotic expression system. The gene products were purified and tested for immunogenicity in ducks.

## Materials and Methods

### Ethics statement

This study was conducted within the Guidelines of Regulations for the Administration of Laboratory Animals (Decree No. 2 of the State Science and Technology Commission of the People's Republic of China on November 14, 1988). All animals used in this study received prior approval from the Hubei Provincial Experimental Animal Manage Committee and Huazhong Agricultural University Academic Committee. The ducklings sampled were anesthetized by intraperitoneal injection with 60 mg/kg sodium pentobarbital. The survival ducks were euthanized with 150 mg/kg sodium pentobarbital at the end of the study.

### Bacterial culture

*R. anatipestifer* strain serotype 1 was from the Pathology Laborayory in the Academy of Agricultural Sciences of Hubei Province, China. *R. anatipestifer* was cultured in tryptic soy broth (TSB; Difco, USA) at 37°C. The bacteria from exponential growth phase (OD_630nm_∼0.5) were split into two culture tubes containing 5 mL TSB medium each. To remove iron from the medium, a final concentration 200 µM of 2, 2′-dipyridyl (Sigma) was added into tube A [16], while tube B was kept 2, 2′-dipyridyl free for the normal control. The bacteria were harvested by centrifugation at 8000 rpm for 10 min after the incubation at 37°C, 220 rpm, for 16 hrs.

### Serum preparations

The sera were prepared as described previously [17] Briefly, 10 healthy 28-day old Cherry-valley ducks were from the Institute of Animal Science, the Academy of Agricultural Science of Hubei Province, China. Each duck was subcutaneously inoculated with 1 mL (5×10^7^ CFU/mL) of *R. anatipestifer* strain serotype 1 suspension on the dorsal neck and boosted thrice every other 2 weeks for twince. The serum was collected from each surviving duck. The titer of each convalescent serum was evaluated using the *R. anatipestifer* strain serotype 1 outer member protein A enzyme-linked immuno-sorbent assays (ELISA) [17]. The convalescent serum with the greatest titer was used in the following study.

### Protein extractions

The protein extraction were prepared as described previously [18] Briefly, the bacteria were cooled on ice for 30 min. A final concentration of 1 mmol/L phenylmethanesulfonyl fluoride (PMSF) (Amersham Biosciences, America) was mixed into each tube. The supernatant was harvested by centrifugation at 6000 rpm, 4°C for 10 min and filtered to remove residual bacteria and bacterial debris by 0.22 µm in diameter filters (Millipore, America). Then a final concentration of 15% trichloroacetic acid (TCA) was added and the mixture was incubated at 4°C for 8 hrs. The precipitate was harvested by centrifugation at 12000 rpm 4°C for 15 min. The pellet was lyophilizedly dried after washing with 0.1% Dithiothreitol (DTT) at −20°C and centrifuging at 12000 rpm 4°C for 15 min. thrice. The pellet was redissolved in Lysis buffer (7 mol/L urea,2 M Thiourea, 4% 3-[(3-Cholamidopropyl) dimethylammonio]-1-propanesulfonate (CHAPS),65 mM DTT) for 2 hrs. The final supernatant was harvested by centrifugation at 12000 rpm 20°C for 15 min and preserved at −80°C. The protein concentration was measured by Bradford Protein Assay Kit (Beyotime, China).

### Protein analyses by 2-D PAGE

The first-dimensional isoelectric focusing (IEF) was performed according to the manufacturer's instructions. Briefly 1 mg of protein sample (micropreparative) was applied on each IPG DryStrips (18 cm, a pH range of 4–7; Amersham Pharmacia Biotech, Sweden). After IEF, each strip was equilibrated in 10 mL equilibration buffer 1 (6 M urea, 0.5% DTT, 30% glycerol, 50 mM Tris-HCl pH 8.8) for 15 min and then in 10 mL equilibration buffer 2 (6 M urea, 4.5% iodoacetamide, 30% glycerol, 50 mM Tris-HCl pH 8.8) for 15 min. For the second dimension, vertical slab SDS-PAGE (12.5%) was run for 4 hrs (30 mA/gel; Bio-Rad protean II Xi, Bio-Rad laboratories, USA). The gels were then stained with ammoniacal silver staining or CBB G-250 from Amresco (Solon, OH, USA). Scanning was carried out with ImageScanner (Amersham Pharmacia Biotech), and image analysis was carried out with version 3.1 of ImageMaster 2D Elite. Up-regulation secreted protein spots were selected by the ratio (≥2) of spot density in iron restriction compared with normal condition [19].

### In-gel protein digestion

Each visible spot on the 2-DE gel was excised, transferred into a well of a 96-well plate, and washed with 50 mL of 25 mM ammonium bicarbonate/50% ACN at ambient temperature for 30 min three times. The samples were shrunk with 50 mL of ACN and re-swollen with 5 mL of 25 mM ammonium bicarbonate containing 10 ng of trypsin at 4°C for 30 min. In-gel tryptic degradation was performed overnight at 37°C, followed by three subsequent extractions. The pooled extracts were lyophilized and reconstituted in 2 mL of 0.1% (v/v) TFA prior to MALDI-TOF MS analysis.

### Mass spectroscopic analyses of proteins

MALDI-TOF MS analysis was prepared according to a previously described procedure [19]. α-Cyano-4-hydroxycinnamic acid(CHCA) was used as the matrix. MALDI-TOF spectra were calibrated using trypsin autodigestive peptide signals and matrix ion signals. MALDI analysis was performed by a fuzzy logic feedback control system (Ultraflex II MALDI TOF/TOF system Bruker, Karlsruhe, Germany) equipped with delayed ion extraction. The peptide mass fingerprint data were submitted to the MASCOT Sequence Query server (http://www.matrixscience.com) for identification against the NCBI database (http://www.ncbi.nlm.nih.gov/BLAST). The criteria used to accept protein identification were based on the data of the peptide mass fingerprint, including the extent of sequence coverage, number of peptides matched and probability score. The sequences of the identified proteins were searched in the BLASTX server (http://www.ncbi.nlm.nih.gov/BLASTX/) to find homologous sequences and putative functions.

### Selection of candidate proteins

The isoelectric point (pI) and molecular mass values of the identified protein spots on 2-DE gels were estimated using the ImageMaster 2D Elite software. Proteins were chosen from all the spots according to the conditions that the estimated pI and mass values were similar with their theoretical pI and mass values. The values of gel-estimated molecular mass of protein Riean_1750 and Riean_1752 marched well to their theoretical predictions. Moreover they were 2 novel proteins with unknown functions and protein family. These two novel proteins were selected for the expressing in prokaryotic expression systems and their immunogenicity in this study.

### Polymerase chain reaction

The total DNA was extracted by Bacterial DNA extraction kit (Omega, America). According to the MS results, the amino acid sequences and DNA sequences of Riean_1750 and Riean_1752 were identified and the gene-specific primers were designed by Primer Premier 5. The primers are (The underlines indicate the restriction sites of endonucleases, *Bam*H and *Xho*): F1750 (5′-CCATGGATCCATGAAAAATATATTTTGG-3′), R1750 (5′-CGCCCTCGAGTTATTTTAAATAAGTTTT-3′); F1752 (5′- CCATGGATCCATGAAAAAAATAACTTAT-3′), R1752 (5′-CGCCCTCG AGTTACGGTAAAATGATTTT-3′).

The DNA sequence encoding the suspect proteins were amplificated by PCR and cloned separately into pET-28a(+) vectors. PCR was carried out according to the manufactural introductions of DNA Thermal Cycler (Biorad, USA) and PCR kit (Invitrogen, Carlsbad, CA, USA) with following conditions: 95C for 5 min; 30 cycles of 95C for 50 sec, 55C for 50 sec, and 72C for 90 sec; a final extension at 72C for 7 min.

### Vector construction, gene cloning and expressing

Vector construction, plasmid transforming and recombinant protein expression were carried out according the descriptions of the manuals (Transgen Biotech, Beijing, China). Briefly, *Escherichia coli* (*E. coli*) strain BL21 (DE3) was used for transforming pET-28a(+) recombinant plasmids. *E. coli* was cultured in Luria Bertani (LB) broth and transformants were selected on media supplemented with 100 mg/mL Kanamycin. The optimal expression of recombinant proteins was achieved by the addition of isopropyl-b-D-thiogalactopyranoside (IPTG) to a final concentration of 1 mM during the mid-exponential growth phase for 5-hour duration.

### DNA sequence analysis

The recombinant plasmids were sequenced in reactions containing approximately 500 ng plasmid DNA. This part of work was finished by Shanghai Sangon Biotech Company, China. Homology searches of the nucleotide sequences were performed using the Blast in the NCBI (http://blast.ncbi.nlm.nih.gov/Blast.cgi).

### Purification of recombinant proteins and plasmids

The above recombinant proteins were extracted in Buffer A (50 mM Tris, pH 8.0, 50 mM NaCl, 0.5 mM EDTA, 0.5% Tween 20) containing 8 M urea (100 mL Buffer A in 1 L of induced bacterial culture). The mixtures were disrupted twice by ultrasonic treatment for 30 sec at Speed 3 and cooled on ice for 15 min. The lysates were centrifuged at 10,000 rpm for 15 min. The supernatants were collected and the pellets were sequentially re-suspended in 20 mL of lysis buffer containing 8 mol/L urea. The purities of the recombinant proteins were measured by using SDS-PAGE, coomassie blue staining, and immunoblotting with *R. anatipestifer* positive duck sera.

### Immunization and challenge

Thirty 1-day-old healthy ducklings were acquired from the Institute of Animal Science, the Academy of Agricultural Science of Hubei Province, China. At seven-day-old, they were then divided into five groups with six ducklings, each our groups were subcutaneously immunized with 500 µg substance as fllows: (1) recombinant Riean_1750 protein, (2) recombinant Riean_1752 protein, (3) recombinant two novel protein mixture, (4) and (5) phosphate buffer solution (PBS), in Freund's complete adjuvant (Sigma) and boosted twice with 500 µg recombinant protein in Freund's incomplete adjuvant (Sigma) on day 14 and day 21. The LD50 of *R. anatipestifer* was 1×10^9^ CFU (data not shown) and the challenge dose was set to double LD50 acording to the previous data [20], [21]. The ducklings of (1),(2),(3),(4) group were challenged by subcutaneous injections with 2 mL inoculum (1×10^9^ CFU/mL) of the serotype strain 1 at the left or right proximal femur on day 28, and the ducklings of (5) group were injected with PBS. The mortality was monitored for 10 days post challenge. The survivals were humanely euthanized at the end of study on day 38. All ducklings including these found dead were necropsied and bacterial cultures of infected organs were preformed.

## Results

### The protein profiles of *R. anatipestifer* were significant different in iron restricted medium

**Figure 1:**
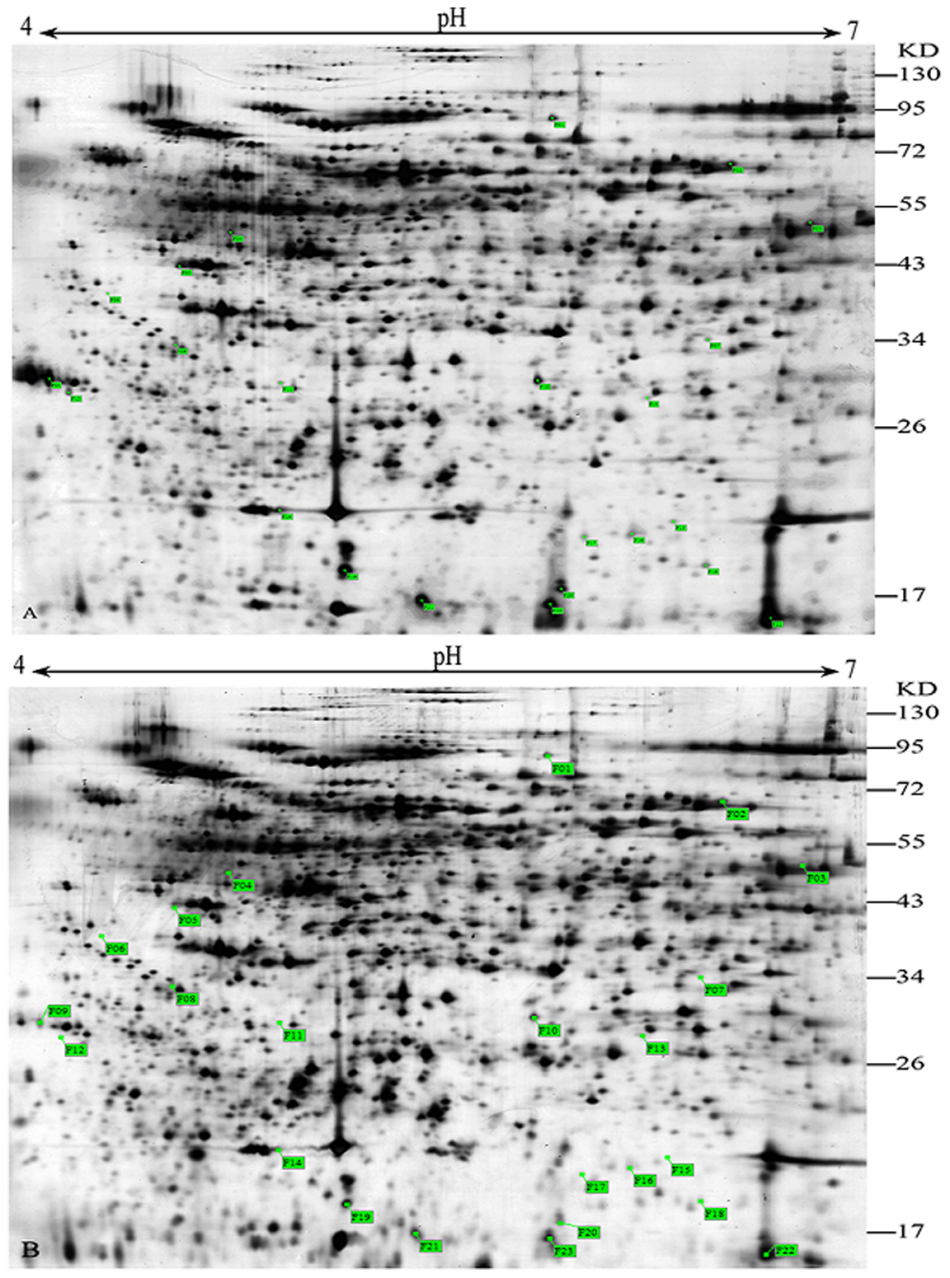
2-DE photograph of secreted proteins of *R. anatipestifer*. Two-dimensional electrophoresis (2-DE) gel of the secreted proteins of *R. anatipestifer* cultured in iron restriction (A) and normal condition (B).

**Figure 2:**
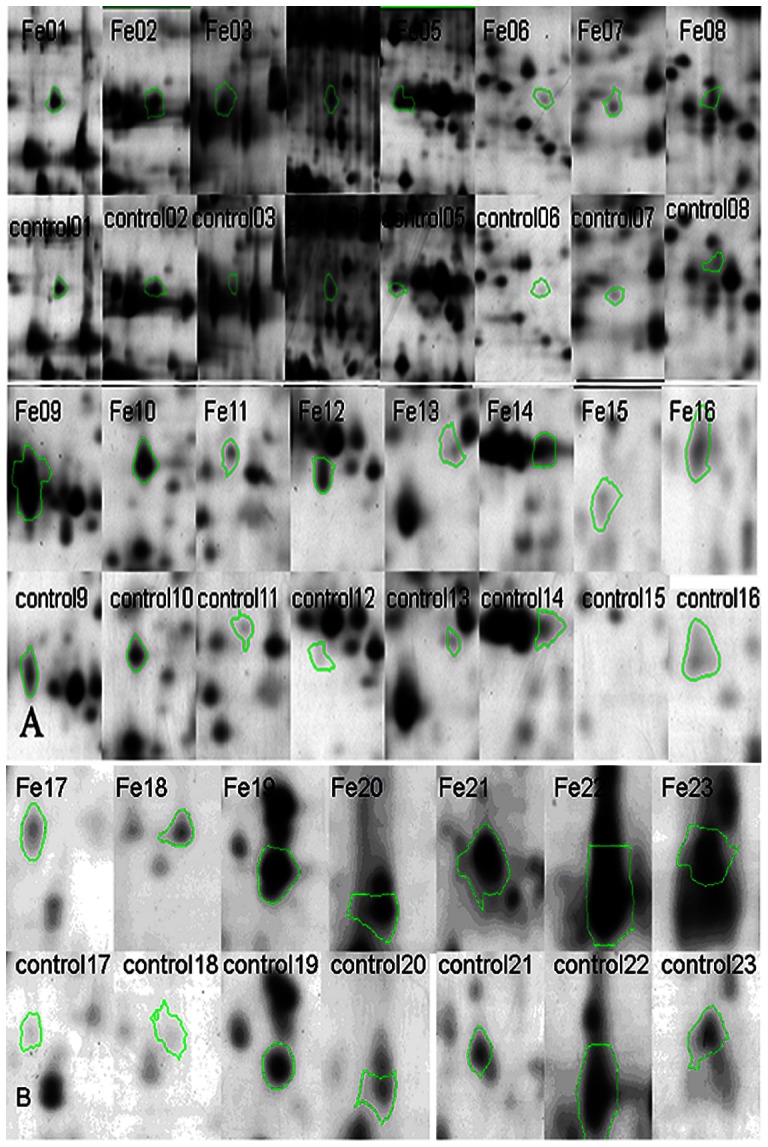
The detail map of the *R. anatipestifer* secreted proteins. spot Fe1-Fe16 by 2-DE in iron restriction (A) and spot Fe17-Fe23 in normal condition (B) noted in color circle.

Total 23 proteins were significantly up-regulated in iron restricted group (Fig.1A and B). Five proteins (Fe06, Fe12, Fe15, Fe17, and Fe18) were detected in iron restricted group, but not detectable in the controls (Fig.2A and B). Eighteen proteins were significant higher expressed in iron restricted group (Fe01, Fe02, Fe03, Fe04, Fe05, Fe07, Fe08, Fe09, Fe10, Fe11, Fe13, Fe14, Fe16, Fe19, Fe20, Fe21 and Fe22) (Fig.2A and B).

### Mass spectroscopic identification and analysis of proteins

Based on the higher ratio (≥2.5) of spot density and molecular weight (MW) range of 17–72 Kd, 12 (Fe01, Fe02, Fe05, Fe08, Fe09, Fe10, Fe17, Fe18, Fe19, Fe21, Fe22 and Fe23) of 23 above proteins from the iron restriction medium were selected for mass spectroscopic analysis. Protein functions were analyzed by in silico analysis of proteins with bioinformatics tools (Mascot software, Matrixscience Company). The functional classifications of these 12 proteins are listed in Table 1. These proteins belong to 6 protein families of *R. anatipestifer* stain DSM 15868, RA-YM, and RA-GD. Fe01 is a fibronectin type iii domain protein. Fe02 is a secreted subtilase family protein. Fe05 is a phosphoglycerate kinase. Fe08 is a translation elongation factor 1a (ef-1a/ef-tu). Fe09 is a leucine-rich repeat-containing protein. Fe17, Fe18, Fe22, and Fe23 are the identical proteins of galactose-binding domain-like protein. Fe10, F19 and F21 are 2 novel proteins (F19 and F21 are identical) with unknown family. The theoretical Mr/pI of Fe10 was 34.8 kDa and 6.02, respectively. And the observation Mr/pI was 40.1 kDa and 5.78, respectively. Mascot score was 506. The theoretical Mr/pI of Fe19 was 23.2 kDa and 5.03, respectively. And the observation Mr/pI was 23.7 kDa and 5.11, respectively. Mascot score was 171. Although the mascot score of Fe19 was not high, the theoretical Mr/pI and observation Mr/pI were almost the same. These 2 novel proteins were designated as Riean_1750 and Riean_1752 and selected for the further function study.

**Table 1:** **The results of selected spots of secreted proteins of** ***R. anatipestifer*** **cultured in iron restriction identified by MS analysis.**

Spot no.	Protein Name	Gi Number	Mascot score	Theoretical Mr/PI	Observation Mr/PI	Sequence Coverage (%)
Fe01	fibronectin type iii domain protein	gi|313206430	339	94.1/6.35	72.4/5.83	11
Fe02	secreted subtilase family protein	gi|315022645	304	62.3/5.91	62.8/6.54	32
Fe05	phosphoglycerate kinase	gi|313206579	241	42.7/4.80	55.6/4.62	27
Fe08	translation elongation factor 1a (ef-1a/ef-tu)	gi|313206993	306	43.3/5.05	47.9/4.55	27
Fe09	leucine-rich repeat-containing protein	gi|313207242	164	40.9/5.17	43.7/4.20	26
Fe10	hypothetical protein Riean_1750	gi|313207234	516	34.8/6.02	40.1/5.78	46
Fe17	Galactose-binding domain-like protein	gi|325335315	133	28.3/9.11	26.7/5.91	18
Fe18	Galactose-binding domain-like protein	gi|325335315	211	28.3/9.11	25.4/6.35	28
Fe19	hypothetical protein Riean_1752	gi|313207236	171	23.2/5.03	23.7/5.11	22
Fe21	hypothetical protein Riean_1752	gi|313207236	93	23.2/5.03	19.9/5.41	25
Fe22	Galactose-binding domain-like protein	gi|325335315	117	28.3/9.11	18.7/6.78	18
Fe23	Galactose-binding domain-like protein	gi|325335315	148	28.3/9.11	18.9/5.82	23

### Expression, purification and analysis of 2 novel proteins

**Figure 3:**
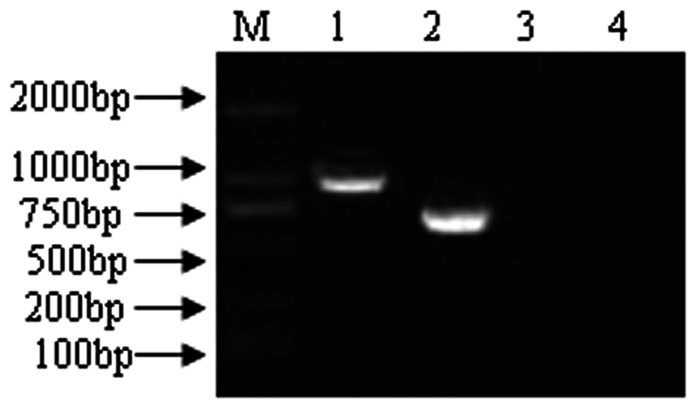
The RCR products of two hypothetical protein gene of *R. anatipestifer*. The Fe10 and Fe19 gene of hypothetical secreted proteins of *R. anatipestifer* cultured in iron restriction. M: DL2000 DNA marker;1: RCR products of Fe10;2: RCR products of Fe19;3: Fe10 negative group;4: Fe19 negative group.

**Figure 4:**
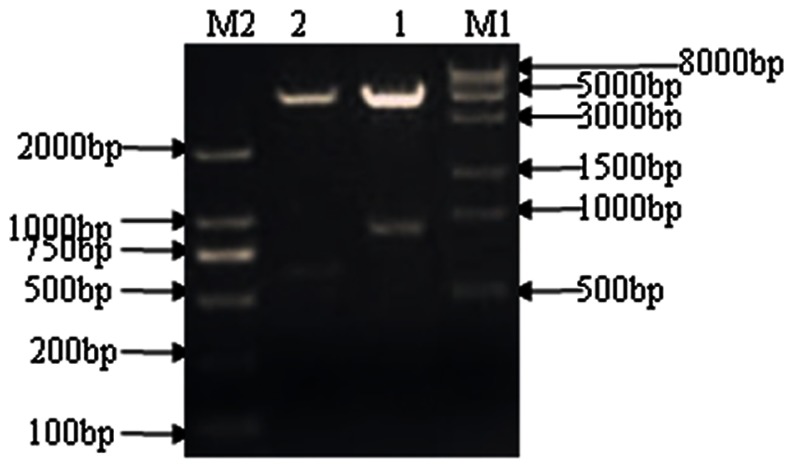
Identification of two hypothetical secreted protein gene of *R. anatipestifer*. Identification of the recombinanted genes of two hypothetical proteins of *R. anatipestifer* cultured in iron restriction pET-28a(+)-Fe10 and pET-28a(+)-Fe19 plasmid with *Bam*HI and *Xho*I. M1: DL8000 DNA marker; M2: DL2000 DNA marker; 1: The double enzyme digest product of pET-28a(+)-Fe10 recombinanted plasmid; 2: The double enzyme digest product of pET-28a(+)-Fe19 recombinanted plasmid.

**Figure 5:**
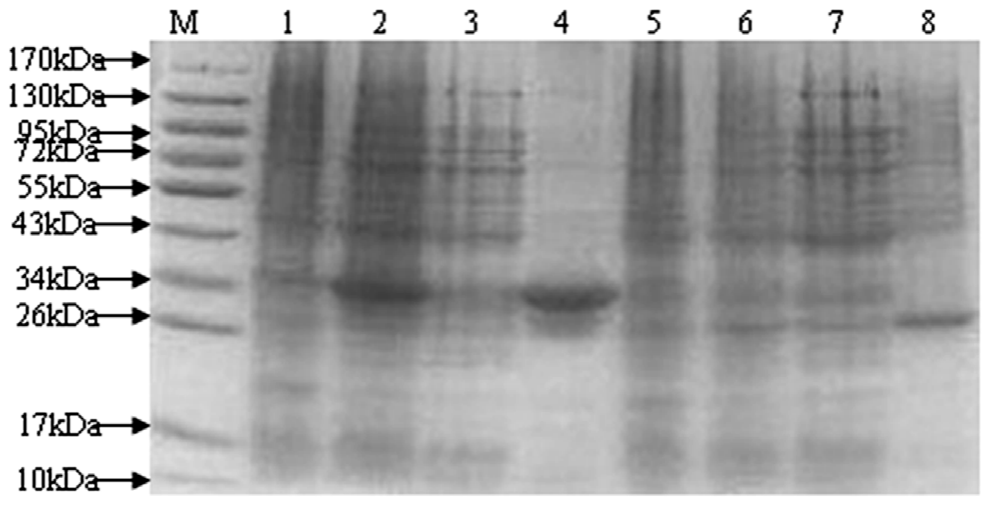
SDS-PAGE of recombinant hypothetical secreted protein of *R. anatipestifer*. M: Protein Marker; 1: The Fe10 protein before induced; 2: The Fe10 protein after induced; 3: Soluble protein after sonication bacilli of Fe10; 4: Precipitat protein after sonication bacilli of Fe10; 5: The Fe19 protein before induced; 6: The Fe19 protein after induced; 7: Soluble protein after sonication bacilli of Fe19; 8: Precipitat protein after sonication bacilli of Fe19.

**Figure 6:**
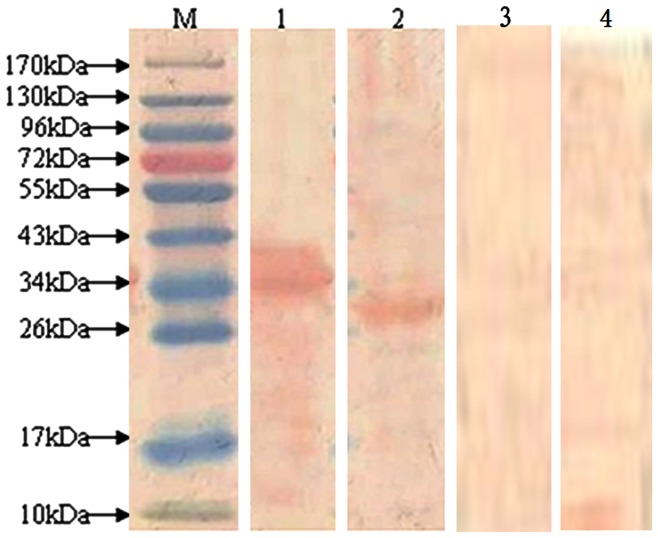
Western-blot analysis of recombinant hypothetical secreted protein of *R. anatipestifer* with duck antisera against *R. anatipestifer*. M:Protein Maker;1:The recombinant protein pET-hypothetical protein Riean_1750 2; The recombinant protein pET-hypothetical protein Riean_1752.

The purified PCR products for novel protein Riean_1750 and 1752 were cloned into pET-28a(+) (Fig.3). The orientation and sequence of the inserts were verified by DNA sequence analysis using primers flanking the insert on the vector as well as internal primers. The fusion proteins pET- hypothetical protein Riean_1750 and 1752 were successfully expressed in *E. coli* strain BL21 (Fig.4). Both pET- hypothetical protein Riean_1750 and 1752 were insoluble and lysed in lysis buffer containing 8 mol/L urea. The molecular weights of pET-hypothetical protein Riean_1750 and 1752 were 34 KDa and 23 KDa, respectively (Fig.5). The specificities of pET-hypothetical protein Riean_1750 and 1752 were confirmed by Western blotting, but no reaction to the serum from *R. anatipestifer* negative ducks (Fig. 6).

### Immune protection of Riean_1750 and 1752

The mortality was summarized in table 2. The ducks immunized with novel protein Riean_1750 and 1752 had a high protection from *R. anatipestifer* challenge. The mortalities of these immunized ducks reduced to 33.3% and 16.7%, respectively. All of 6 ducks immunized with a mixture combined with equal amount of Riean_1750 and _1752 were survived from *R. anatipestifer* challenge. Necropies were performed for all animals. The dead ducks showed classic serositis,and *R. anatipestifer* were isolated from their organs in any infected ducks. The control ducks of (5) group were normal during the study.

**Table 2:** **Mortality of ducks immunized by recombinant** ***R. anatipestifer*** **secreted proteins when challenged with a virulent** ***R. anatipestifer*** **strain serotype 1.**

Group No.	Immunization	Challenge	No.of ducks	No. of deaths (days after challenge)										No. of death (total)	Mortal-ity (%)
				1	2	3	4	5	6	7	8	9	10		
(1)	hypothetical protein Riean_1750	+	6	0	1	1	0	0	0	0	0	0	0	1(6)	33.3
(2)	hypothetical protein Riean_1752	+	6	0	0	0	1	0	0	0	0	0	0	1(6)	16.7
(3)	hypothetical protein Riean_1750 and hypothetical protein Riean_1752	+	6	0	0	0	0	0	0	0	0	0	0	0(6)	0
(4)	None	+	6	0	2	3	0	1	0	0	0	0	0	6(6)	100
(5)	None	−	6	0	0	0	0	0	0	0	0	0	0	0(6)	0

Note: + indicates that ducks were challenged; − indicates that ducks were not challenged.

## Discussion

In this study, 23 proteins from *R. anatipestifer* cultured in the iron depletion medium were identified. Twelve of 23 proteins were analyzed by mass spectrography. Of them, 9 of 12 proteins belong to 6 different protein families, while other 3 (Fe10, 19 and 21) are novel proteins. Among the 12 proteins which were identified by mass spectrography, Fe10 and Fe19 were chosen to identify their immunogenicity. Based on their theoretical Mr/pI and observation Mr/pI were similar and their mascot score were high. Novel protein Fe19 and Fe21 are identical. Fe10 and Fe19 were focused in this study because their functions are uknown. They may have the important roles in the mechanism of bacterial survival and possible potential for bacterial diagnosis and vaccination. Our data demonstrated that the novel protein Riean_1750 and 1752 had high immunogenicity that the mortalities of ducks decreased to 33.3% and 16.7%, respectively, while the ducks immunized with a mixture of Riean_1750 and 1752 had 100% protection from *R. anatipestifer* challenge. In vivo Fe^2+^ was not enough to support the antigen to reproduction [10], [11]. When one antigen attacks the host, the pathogen will enhance expression of some virulence factors. And the up-regulated proteins may be the virulence factors which may express lower in normal conditions [10], [11].

The Fibronectin type III domain (Fe01) is an evolutionary conserved protein domain that is widely found in animal proteins. The fibronectin protein in which this domain was first identified contains 16 copies of this domain. The domain is about 100 amino acids long and possesses a beta sandwich structure. Fibronectin domains are found in a wide variety of extracellular proteins. They are widely distributed in animal species, but also found more sporadically in yeast, plant and bacterial proteins [19], [22], [23].

Fe02, a secreted subtilase family protein, may be a virulence factor of *R. anatipestifer*. The mechanism of its increased expression in this study is unknown. Its expression in prokaryotic expression system was unsuccessful (data not shown). Phosphoglycerate kinase (Fe05) is a transferase enzyme involved in glycolysis. A higher phosphoglycerate kinase in iron restricted medium may facilitate ATP synthesis by transfering phosphate groups from 1,3-bisphosphoglycerate to ADP[24].

Fe17, Fe18, Fe22 and Fe23 are identical, a protein structural motif that forms a α/β horseshoe fold [25], [26]–[28].

Fe08, one of translation elongation factors, facilitates translational elongation in protein synthesis. Its high expression suggested that *R. anatipestifer* is able to enhance its own translation system, as a defense mechanism of resistance to harsh environment[29].

DPD (2,2′-dipyridyl) has been widely used to reduce the iron concentration in the medium [16], [30]-[32]. DPD has a six-membered cyclic structure that can decrease the free iron ions by forming stable chelates with iron in the culture medium. DPD treated medium has a low iron environment that is similar to that in the body of bacterial infected animals [30]. To adapt to an environment of iron depletion, bacteria respond by expressing a large numbers of genes that can up-regulate the uptake of iron from the environment [33]. 2-D PAGE is a common technique for the protein separation and comparison [22]. 2-D PAGE was repeated twice and the results were consistent in this study.

In summary, 2 novel proteins were identified from *R. anatipestifer* cultured in iron restricted medium. They are insoluble proteins with molecular weights of 34 KDa and 23 KDa, respectively. The immunogenicity study demonstrated that that ducks received high protection when the ducks immunized by either one of these 2 proteins. There was a 100% protection rate when the ducks immunized by the combination of these 2 proteins.
